# Caution and Simplicity, the True Test of Excellence in the Practice of Medicine

**Published:** 1867-10

**Authors:** 


### EDITORIAL AND MISCELLANEOUS.

### CAUTION AND SIMPLICITY, THE TRUE TEST OF EXCELLENCE IN THE PRACTICE OF MEDICINE.

History repeats itself. Thirty years ago many of the schools and very many of the first minds engaged in teaching and practicing medicine, were swayed with a love ot the heroic, and, abandoning conservatism, set about demolishing the whole phalanx of morbid phenomenon with as little ceremony as if it were fun. Sixty grains of Calomel was the minimum portion, the maximum was an ounce, or what you pleased. Blood was taken by the quart or half gallon, and I have seen ten blisters doing the work of counter iritation at one time on a single subject. Salivation, sloughing, loss of teeth, and other hard consequences followed. Tartar emetic and Cook's Pills, occupied a prominent place in the foreground. Calomel was called the Sampson, and Tartar Emetic the Herculean club of the Materia Medica, while the lancet, blisters and Cook's pills might have been dubbed, with propriety, the battle axe, scalping knife and shot gun. As a consequence of this propagandism, conservatism was crushed out. None but heroes found employment, radical treatment was the order of the day, for everything that smacked of *morbid action.* Fees were doubled, and fortunes were to be made in a year or so. But the public mind was not tranquil, a revolution was impending, and it came. Our heroes were all banished to rural shades, without time to enquire why or wherefore. Thompsonianism, like Minerva from the brain of Jove, stood before the whole country, from Boston to Savannah, in full manhood, proclaiming that every drug in the Materia Medica was an enemy to human life, and that bleeding would certainly kill. Their theory was simple, "heat is life and cold is death." *Take* the "canker" out of the stomach with Cobelia, and fill it with red pepper to keep the man warm, and he is safe until old age garners him home, free from pain, and his business all settled up.—As a consequence every man who had lost a member of his family, or his teeth, or was bald headed, sore eyed, thin visaged, or ugly, attributed it to the faculty, and swore worse than the "army did in Flanders," that they should doctor him no more. And many have kept their word. Thomsonianism is a living heresy, ridiculous as it is, and will continue another generation or two, in all probability. But this is not the only heresy which the grand old profession of Medicine, in its moments of quixotism, has allowed to rise up and become formidable. Hydropathy, founding its claims upon the curative effects of water, has bounded into life, dating no further back than the early part of the nineteenth century, and yet claiming to be the first to discover its healing power. Notwithstanding its uses date back to the great monarchies of Babylon, Assyria and Egypt. Notwithstanding it was practiced by Hypocrates five hundred years before Christ, and by Galen in the days of the Roman Commonwealth, and by wise men of the profession ever since. Still this monstrous absurdity, with not a semblance of truth to back it, from the dependence of the profession upon other things, and their neglect to enforce its frequent use, as one of the greatest adjuncts we have in the treatment of fevers and inflamations, has permitted a system founded upon this alone as a curative, to take root and become formidable over the whole world.

Homopathy is such a dear little harmless thing, that we scarcely know how to characterize it. It is the "expectant system," Cullan called it the expectant plan, that is, in simple and doubhtful cases, to wait, let the simple get well and the doubtful ones develope. Hahnemann was a smart fellow he makes a "system" out of it, and the children must have the little pelletts of goat's milk and sugar, or there will be no doing anything with them. We will relate an anecdote: In the city of ——, Ky., a couple of polished gentlemen from the "Hub," located as Homeophetic physicians. A wealthy gentleman had a little excentric daughter, six years old, whom he was anxious to have cured of her excentricities.—He had consulted every medical man in reach, without any benefit. He speedily made the acquaintance of the two nice gentlemen named. He asked their advice, and secured their services. They examined the case and assured the anxious parents that the child could be radically cured in a few months. Taking from his very handsome pocket case two little bottles, filled with pellets, about the size of mustard seed, he ordered one before each meal from one bottle, and one after each meal from the other, but warned them of the great danger of reversing the order, or giving more than one of each at a time. And further, if any unpleasant symptom should follow, to inform him promptly, but out of abundant caution, he consented to call night and morning with the antidote, and guard the case at all points. Two or three days passed, the child rejoiced when the time came to take the medicine and always wanted more. The bottles were kept out of sight on the fire board. It happened that all were out of the house but the child, by the aid of a chair, it reached both bottles, and eat up their entire contents, and when the mother returned, was playing with them as toys. The story was soon told, the mother fainted, all the Doctors in town were sent for, the two Homeopathics amonst them. The father called for the antidote, the Doctor had forgotten what was the antidote. The child laughed and frolicked as though nothing was wrong, called for more medicine. The scene beggered description. Homeopathy was stumped for once. The patron, a fine gentleman, concluded to give over any further efforts at curing the child. But neither of the parties liked the joke.

Now none of these vagaries ought ever to have had a stage of incubation, much less to have hatched, and grown to power and influence, and certainly they never would have done so, if the profession had been true to itself. The fault lies, not in the profession, but its friends. Not in its truths, but the bad use that is made of them. Nor are the rank and file to blame, they are generally earnest men closely engaged with their duties as they have been taught them. But there are men in the profession who are talented, educated, plausible and ambitious, who talk well and write well, but have little taste for the drudgery, as represented in the detail of an honest, night and day, rough and tumble practice, who love to sleep, and see much of society, and convivial life, and yet the means of doing all this has to be improverised out of the daily gleanings of their profession—specifics are thought of. Some grand idea of beating the "tiger," by which fame is to be won, or notoriety at least, sufficient to keep off drudgery and keep the supply bills paid. The profession of Medicine has never been injured by its enemies, they have never been sufficiently powerful to do it hurt. Its greatest injuries have been inflicted by its professed friends. The public mind, ever awake to its own interest, is constantly inquiring how the matter stands in medicine. Thompson, Haunemann, Priesnitz (I believe is his name, and if I have missed it, or the arthography is at fault, I beg pardon) "we loose no patients," says this tryo, "not a man has died under our treatment since the profession, as practiced by us, began its briliant career." And yet people die, whose patients were they ? Not the patients of our bastard cousins, for they never loose any, they were undoubtedly the patients of the faculty, for they never defend themselves against the charge of killing every body that dies. Now the plain truth is simply this, the public are willing to trust to these men until they become seriously ill—often when beyond the reach of remedies—our cousins are dismissed or quit the field, as we have many times observed them to do, and some medical gentlemen is called, and if the man dies the death is charged to the profession. Hence in the great Metropolis of England, the premium on life insurance is smaller where Homeopathists have charge of the health and life, than where the regular profession have it, (with certain companies, I mean.) They are able to prove that under their treatment nobody dies.—Whilst Sir Thomas Watson and Tilbury Fox, and the great minds of London, would scorn to dispute an assumption, not claimed by their equals, nor founded on a single fact even, but the mere catch word of a jockey, caught up and repeated by the crowd, who know nothing, and can know nothing, of the merits of the case.

A late writer on Homeopathy, says wittily, there is in it nothing true that is new, and nothing new that is true.

As we have said before, all these systems have resulted from the haste of some of our brightest men to gain notoriety—to become leaders and propagandists, and be gazetted all over the world as the author of something new. McCauley says, (see Miscellanies) that the attraction of gravitation would have been discovered just when it was, if the great Newton had never lived, that great discoveries come in the natural course of experience, and requires only the earnest use of such well established truths as we have, to develope others. The gold hunter first finds the coveted treasure in grains, then in pennyweights and finally, perhaps, nuggets, but this does not establish the value of the mine, he goes on until he finds the vein, the great matrix, before he exclaims Eruka.

It would be well if Medical men would imitate this prudence. We have developed ideas enough, and fast enough. We have a plenty of the raw material, the thing we want is patient, earnest thinkers to study facts, and trace out their leadings, until great embryo truths, as yet hidden from view, are laid bare alike to the eye of the believer and skeptic. To effect this, let the profession come back to first principles. Simplicity is nature's law, embrace it, study it in this way, and at every step she will unfold new beauties and new treasures. What a field is open to us in the nervous system, the circulation, the laws that govern the functions, organic election, &c., &c. Much as we have achieved in Medicine, we have scarcely approached the shores of the great ocean of truth.—Let us not run away with a single idea, and delude ourselves with hypotheses, but awake to the true dignity and responsibility of our high calling, and labor for truth alone.

But we fear that history is about to repeat itself, notwithstanding the severe lessons of the past. While cautious men are speculating upon the danger attending the use of chloroform, and, by a close analysis of its intricate chemical constitution, to ascertain upon what that danger depends, it has been as thoroughly utilitised by some of our enterprising co-laborers, as paregoric or salts. Classed amongst the sedative narcotics, it is claimed by some, that it acts through the nervous system upon the organism. Other theories are extant maintaining that its action is directly upon the blood, and sharing the properties of all other narcotics, may be given with equal safety. We dissent entirely from this latter opinion, and warn our heroic friends to beware of its careless inhalation.

